# A Salmon Protein Hydrolysate Exerts Lipid-Independent Anti-Atherosclerotic Activity in ApoE-Deficient Mice

**DOI:** 10.1371/journal.pone.0097598

**Published:** 2014-05-19

**Authors:** Cinzia Parolini, Rita Vik, Marco Busnelli, Bodil Bjørndal, Sverre Holm, Trond Brattelid, Stefano Manzini, Giulia S. Ganzetti, Federica Dellera, Bente Halvorsen, Pål Aukrust, Cesare R. Sirtori, Jan E. Nordrehaug, Jon Skorve, Rolf K. Berge, Giulia Chiesa

**Affiliations:** 1 Department of Pharmacological and Biomolecular Sciences, Università degli Studi di Milano, Milan, Italy; 2 Department of Clinical Science, University of Bergen, Bergen, Norway; 3 Research Institute of Internal Medicine, Rikshospitalet University Hospital, Oslo, Norway; 4 National Institute of Nutrition and Seafood Research, NIFES, Bergen, Norway; 5 Department of Heart Disease, Haukeland University Hospital, Bergen, Norway; University of Padova, Italy

## Abstract

Fish consumption is considered health beneficial as it decreases cardiovascular disease (CVD)-risk through effects on plasma lipids and inflammation. We investigated a salmon protein hydrolysate (SPH) that is hypothesized to influence lipid metabolism and to have anti-atherosclerotic and anti-inflammatory properties. 24 female apolipoprotein (apo) E^−/−^ mice were divided into two groups and fed a high-fat diet with or without 5% (w/w) SPH for 12 weeks. The atherosclerotic plaque area in aortic sinus and arch, plasma lipid profile, fatty acid composition, hepatic enzyme activities and gene expression were determined. A significantly reduced atherosclerotic plaque area in the aortic arch and aortic sinus was found in the 12 apoE^−/−^ mice fed 5% SPH for 12 weeks compared to the 12 casein-fed control mice. Immunohistochemical characterization of atherosclerotic lesions in aortic sinus displayed no differences in plaque composition between mice fed SPH compared to controls. However, reduced mRNA level of *Icam1* in the aortic arch was found. The plasma content of arachidonic acid (C20∶4n-6) and oleic acid (C18∶1n-9) were increased and decreased, respectively. SPH-feeding decreased the plasma concentration of IL-1β, IL-6, TNF-α and GM-CSF, whereas plasma cholesterol and triacylglycerols (TAG) were unchanged, accompanied by unchanged mitochondrial fatty acid oxidation and acyl-CoA:cholesterol acyltransferase (ACAT)-activity. These data show that a 5% (w/w) SPH diet reduces atherosclerosis in apoE^−/−^ mice and attenuate risk factors related to atherosclerotic disorders by acting both at vascular and systemic levels, and not directly related to changes in plasma lipids or fatty acids.

## Introduction

Cardiovascular disease (CVD) is responsible for approximately 16–17 million deaths annually, making it the leading cause of mortality in Western countries [1], [2]. The disease encompasses conditions such as coronary artery disease, carotid and cerebral atherosclerotic disease and peripheral artery atherosclerosis resulting in chronic and acute ischemia in affected organs. The underlying pathological process is lipid accumulation leading to atherosclerosis, a slowly progressing chronic disorder of large and medium-sized arteries that can lead to intravascular thrombosis with subsequent development of complications like myocardial infarction (MI), stroke and acute ischemia of the limb [3]. In the last years, inflammation has emerged as an additional key factor in the development of atherosclerosis and seems to be involved in all stages, from the small inflammatory infiltrate in the early lesions, to the inflammatory phenotype characterizing an unstable and rupture-prone atherosclerotic lesion [4]. In fact, today atherosclerosis is regarded as a disorder characterized by a status of non-resolved inflammation, with bidirectional interaction between lipids and inflammation as a major phenotype. Inflammation in atherosclerosis leads to activation of endothelial cells, enhanced expression of adhesion molecules, inflammatory cytokines and macrophage accumulation.

Liver is the main organ regulating lipid metabolism, affecting blood lipids, especially plasma triacylglycerols (TAG) [5]. Recently, investigators have suggested that the liver plays a key role in the inflammatory state of an individual [6], [7], and that dietary cholesterol absorbed by the liver contributes to inflammation [8]. Research into atherosclerosis has led to many compelling discoveries about the mechanisms of the disease and together with lipid abnormalities and chronic inflammation, oxidative stress has a crucial involvement in the initiation and progression of atherosclerosis [9].

Improvement of life style and dietary habits can reduce some risk factors such as high levels of low density lipoprotein (LDL)-cholesterol, TAG and inflammatory molecules [10]. Fish consumption is consider health beneficial as it lowers plasma lipids and attenuates inflammation [11]. This is linked to the long-chained n-3 polyunsaturated fatty acids (PUFA) content, in particular eicosapentaenoic acid (EPA) and docosahexaenoic acid (DHA). However, fish protein is a rich source of bioactive peptides with valuable nutraceutical and pharmaceutical potentials beyond that of n-3 PUFAs [11]. Fish protein hydrolysates are generated by enzymatic conversion of fish proteins into smaller peptides, which normally contain 2–20 amino acids. In recent years, fish protein hydrolysates have attracted much attention from food scientists due to a highly balanced amino acid composition, as well as the presence of bioactive peptides [12]. The organic acid taurine is mainly found in marine proteins, and is suggested to induce cholesterol-lowering effect by increasing excretion through bile, thus potentially exerting an anti-atherosclerotic effect [13]. Recent studies show TAG-lowering effects [14], [15], antioxidant capacity [12], antihypertensive [11] and cholesterol-lowering effects [16], [17], and potential to reduce markers of reactive oxygen species [18] from fish protein. Therefore, fish protein hydrolysates have been implicated in several processes with potential anti-atherogenic effects. In this study, we examined the anti-atherosclerotic potential of a salmon protein hydrolysate (SPH) on atherosclerotic development in apolipoprotein E-knockout (apoE^−/−^) mice.

## Materials and Methods

### Experimental Design

The study was conducted according to national (D.L. 116, G.U. Suppl. 40, February 18, 1992, Circolare No. 8, G.U July 1994) and international laws and policies (EEC Council Directive 2010/63, September 22, 2010: Guide for the Care and Use of Laboratory Animals, United States National Research Council, 2011). The Italian Ministry of Health approved the protocol (n° 04/2012).

24 female apoE^−/−^ mice from the breeding strain C57BL/6, 8 weeks old, were purchased from Charles River Laboratories (Calco, Italy), and kept under standard laboratory conditions (12 hours light cycle, temperature 22±1°C, humidity 55±5%), with free access to standard chow and tap water. After 1 week of acclimatization under these conditions, mice were randomly divided into two groups of 12 mice. Although apoE^−/−^ mice spontaneously develop atherosclerosis, both groups were fed a high-fat diet (23.7% w/w) consisting of 21,3% lard (Ten Kate Vetten BV, Musselkanaal, Netherlands) and 2.4% soy oil (Dyets. Inc., Betlehem, PA, USA) to accelerate the atherosclerotic formation. The control diet contained 21% w/w casein as protein source, whereas 5% casein was replaced with an equal amount of salmon protein hydrolysate (SPH) (Marine Bioproducts, Storebø, Norway) in the intervention diet. The SPH was produced by enzymatic hydrolysis from salmon by-products (spine) using controlled autolysis with an alkaline protease and a neutral protease, and the resulting protein hydrolysate was then subjected to a second enzymatic treatment with an acid protease A. The final hydrolysate was fractionated using micro- and ultra- filtration and the size distribution of the peptides was analysed. The final preparation consisted of peptides <1200 Da and 25% of the peptides were below 200 Da. The diets were isocaloric containing 21% protein, 24% fat, 42% carbohydrates and 6% micronutrients, and administered for 12 weeks. Other diet ingredients were from Dyets. Inc., and the full composition of the diets, as well as amino acid composition, is given in **Table S1**.

### Harvesting of Tissue

During the treatment period, blood samples were collected at day 1 and after 77 days from the retro-orbital plexus into tubes containing 0.1% (w/v) EDTA after an overnight fast. Blood samples were chilled on ice for at least 15 minutes and stored at −80°C until analyses.

After 12 weeks of treatment, mice were sacrificed under general anaesthesia with 2% isoflurane (Forane, from Abbot Laboratories Ltd, Illinois, USA) and blood was removed by perfusion with phosphate-buffered saline (PBS). Aorta was rapidly dissected from the aortic root to the iliac bifurcation, periadventitial fat and connective tissue was removed as much as possible. Aorta was longitudinally opened pinned flat on a black wax surface in ice-cold PBS, photographed unstained [19] for subsequent plaque quantification (see *En face analysis*), and then immediately put in a tissue-freezing medium, snap-frozen in liquid nitrogen and stored at −80°C. For histological/immunohistochemical analysis, six hearts from each group were removed, fixed in 10% formalin for 30 min and transferred into PBS containing 20% sucrose (w/v) overnight at 4°C before being embedded in OCT compound (Sakura Finetek Euope B.V., Alphen aan den Rijn, The Netherlands) and stored at −80°C. An equal subset of hearts and all livers were immediately snap-frozen in liquid nitrogen for subsequent analyses.

### En Face Analysis

Aorta images were recorded with a stereomicroscope-dedicated camera (IC80 HD camera, MZ6 microscope, Leica Microsystems, Germany) and analysed using ImageJ image processing program (http://rsb.info.nih.gov/ij/). An operator blinded to dietary treatment quantified the atherosclerotic plaques.

### Aortic Sinus Histology/immunohistochemistry

Serial cryosections (7 µm thick) of the aortic sinus were cut. Approximately 25 slides with 3 cryosections/slide were obtained, spanning the three cusps of the aortic valves. Every fifth slide was fixed and stained with hematoxylin and eosin (Bio-Optica, Milano, Italy) to detect plaque area. Plaque area was calculated as the mean area of those sections showing the three cusps of the aortic valves. Adjacent slides were stained to characterize plaque composition. Specifically, Masson’s Trichrome (04-010802, Bio-Optica, Milano, Italy) was used to detect extracellular matrix deposition and Oil red O staining (Sigma-Aldrich, St. Louis, MO, USA) was used to detect intraplaque neutral lipids.

Macrophages and T-lymphocytes were detected using an anti-F4/80 antibody (ab6640, Abcam, Cambridge, UK), and an anti-CD3 antibody (ab16669, Abcam, Cambridge, UK), respectively. A biotinylated secondary antibody was used for streptavidine-biotin-complex peroxidase staining (Vectastain Abc Kit, Vector Laboratories, Peterborough, UK). 3,3′-Diaminobenzidine was used as chromogen (Sigma-Aldrich, St. Louis, MO, USA), and sections were counterstained with hematoxylin (Gill’s Hematoxylin, Bio-Optica, Milano, Italy). To acquire and process digital images an Aperio ScanScope GL Slide Scanner (Aperio Technologies, Vista, CA, USA), equipped with a Nikon 20×/0.75 Plan Apochromat objective producing a 0.25 µm/pixel scanning resolution with a 40× magnification and the Aperio ImageScope software (version 8.2.5.1263) was used. A blinded operator to the study quantified plaque area, extracellular matrix and lipid deposition, as well as inflammatory cell infiltrate. The amount of extracellular matrix, lipids, macrophages and T-lymphocytes was expressed as percent of the stained area over the total plaque area.

### Plasma Lipid and Fatty Acid Composition Measurements

Enzymatically measurements of plasma lipids were performed with an automated method for direct measurement of lipids on a Hitachi 917 system (Roche Diagnostics GmbH, Mannheim, Germany) using triacylglycerol (GPO-PAP), total- and free cholesterol kits (CHOD-PAP) from Roche Diagnostics, and phospholipids FS kit and a non-esterified fatty acids (NEFA) kit from DiaSys (Diagnostic Systems GmbH, Holzheim, Germany). Total plasma fatty acid composition was analyzed as previously described [20].

### Gene Expression in Liver, Heart and Aorta

Total cellular RNA was purified from 20 mg liver, total homogenized heart and pooled aorta samples from six mice using the RNeasy kit and the protocol for purification of total RNA from animal cells and fibrous tissue (Qiagen GmbH, Hilden, Germany), as described by Vigerust *et al*. and Strand *et al*., respectively [21], [22]. cDNA was obtained as described by Strand *et al.* [22]. Real-time PCR was performed on an ABI prism 7900 H sequence detection system (Applied Biosystems, Foster City, CA, USA) using 384-well multiply PCR plates (Sarstedt Inc., Newton, NC, USA) and probes and primers from Applied Biosystems, Foster City, CA, USA as described by Strand *et al*. [22]. The primers used are listed in **Table S2**. Six different reference genes were included for liver: 18*s* (Kit-FAM-TAMRA (Reference RT-CKFT-18s)) from Eurogentec (Seraing, Belgium), ribosomal protein, large, P0 (*Rplp0*, AX-061958-00-0100), hypoxanthine guanine phosphoribosyltransferase 1 (*Hprt1*, AX-045271-00), ribosomal protein, large, 32 (*Rpl32*, AX-055111-00), polymerase (RNA)II(DNA directed) polypeptide A, (*Polr2a,* AX-046005-00) and TATA-box binding protein (*Tbp*, AX-041188-00) all five from Thermo Fisher Scientific Inc. (Waltham, MA, USA). For the heart *18s*, *Rplp0* and *Hprt1* were used, and for aorta *18s*, *Rplp0*, *Rpl32* and *Hprt1*. The software GeNorm (http://www.gene-quantification.de/hkg.html) was used to evaluate the reference genes, and data normalized to *Rplp0* and *Rpl32* for liver, *Hprt1* for heart and *Rplp0* and *Hprt1* for aorta, are presented.

### Hepatic Enzyme Activities

Livers were homogenized and the post-nuclear fraction isolated as described earlier [23]. The assay for carnitine palmitoyltransferase (CPT)-2 was performed according to Bremer [24] and Skorve *et al*. [25], but with some modifications: the reaction mix contained 17.5 mM HEPES pH 7.5, 52.5 mM KCl, 5 mM KCN, 100 mM palmitoyl-CoA and 0.01% Triton X-100. The reaction was initiated with 100 µM [methyl-14C]-L-carnitine (1100 cpm/ηmol), and 35 µg total protein was used. Palmitoyl-CoA oxidation was measured in the post-nuclear fraction from liver as acid-soluble products [26]. The activity of fatty acyl-CoA oxidase (ACOX)-1 and acyl-CoA: cholesterol transferase (ACAT) were measured in post-nuclear fractions as described by Madsen *et al.* [26] and Field *et al*. [27], respectively.

### Measurements of Plasma Inflammatory Markers

Levels of interleukin (IL)-1β, IL-6, IL-10, tumor necrosis factor (TNF)-α and granulocyte-macrophage colony-stimulating factor (GM-CSF) were analyzed on plasma samples collected at day 77 of treatment by Multiplex suspension technology using a customized Bio-Plex Pro Mouse assay (Bio-Rad Laboratories, Hercules, CA).

### Statistical Analysis

The results are presented as mean with standard deviation (SD) for 4–12 mice per group. Normal distribution was assessed by the Kolmogorov-Smirnov test. Unpaired Student’s *t*-test was used to evaluate statistical differences between groups; Mann-Whitney test was applied when data were not normally distributed. A value of P<0.05 was considered statistically significant. Statistical analyses were performed using Prism Software (GraphPad Prism version 5.0; GraphPad Prism, San Diego, CA, USA).

## Results

### The SPH-diet Decreased Atherosclerotic Plaque Development

**Figure 1:**
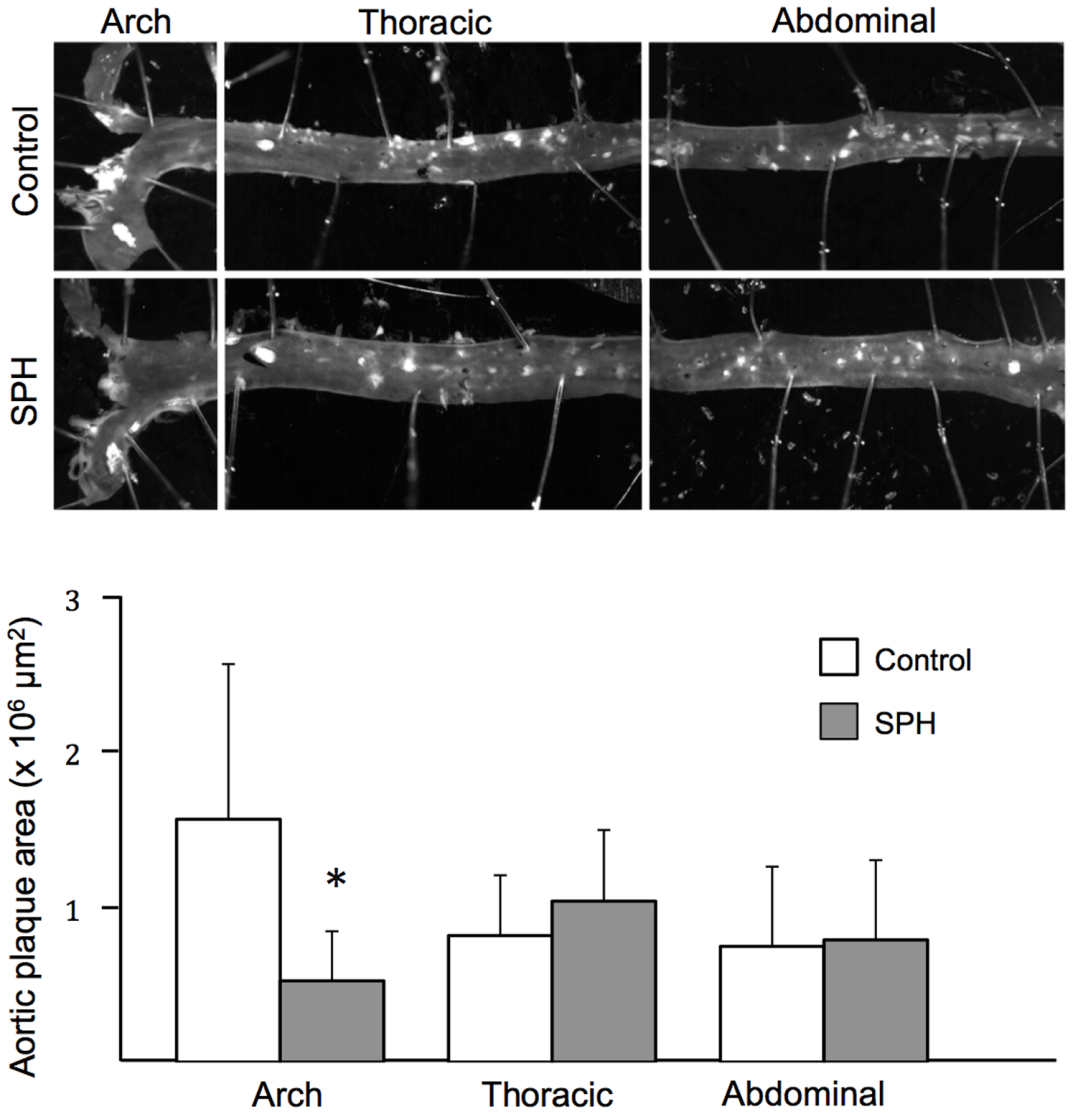
Atherosclerotic plaque level in apoE^−/−^ mice fed a high-fat diet (control) or a diet with 5% SPH. After 12 weeks of dietary treatment, whole aorta was collected and *en-face* analysis was performed to quantify aortic surface covered by atherosclerotic plaques. Bars represent means ± SD of 12 mice for each diet. Unpaired *t*-test was used to detect statistical significance (*P<0.05).

After 12 weeks on a high-fat diet, 5% SPH-fed mice displayed a weight gain similar to the control group. At sacrifice, the average weight gain was 5.98±1.78 g (mean ± SD) in controls and 5.04±0.88 g in SPH mice (P>0.05). A significantly lower plaque development was observed in the aortic arch in SPH-fed mice compared to control mice (0.55±0.33 vs. 1.63±0.99×10^6^ µm^2^; **Fig. 1**, corresponding to 0.91±0.55 vs. 2.72±1.72% of the aortic surface covered by plaque). There were no differences in thoracic (1.08±0.47 vs. 0.85±0.41×10^6^ µm^2^; **Fig. 1**, corresponding to 1.71±0.84 vs. 1.41±0.68% of the aortic surface covered by plaque) or abdominal aorta sections (0.81±0.53 vs. 0.78±0.53×10^6^ µm^2^; **Fig. 1**, corresponding to 1.36±0.89 vs. 1.29±0.88% of the aortic surface covered by plaque).

**Figure 2:**
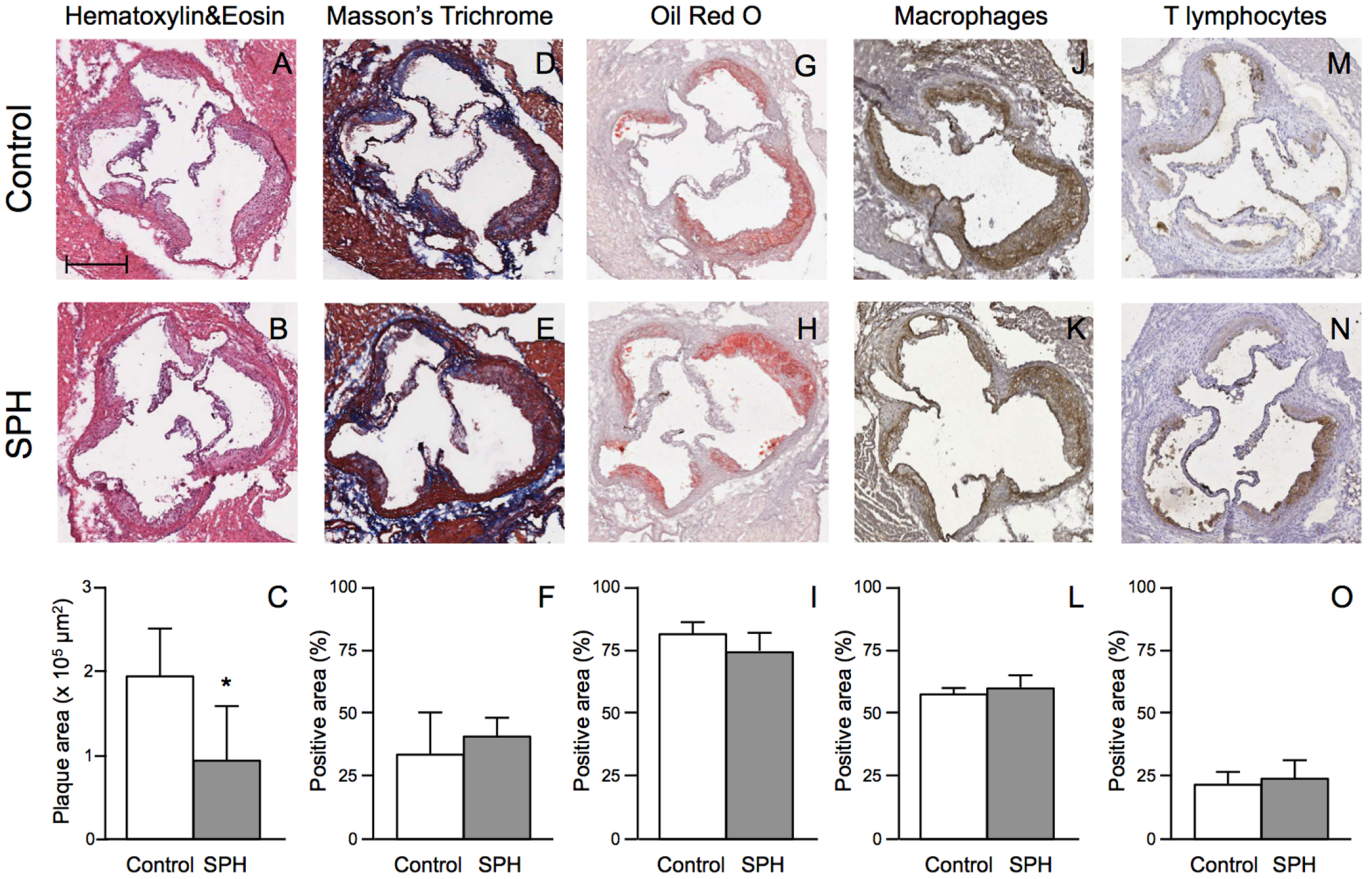
Histological and immunohistochemical characterization of plaques in the aortic sinus in apoE^−/−^ mice fed a high-fat diet (control) or a diet with 5% SPH for 12 weeks. Representative photomicrographs and quantification of maximum plaque area (panels A–C). Representative photomicrographs and quantification of extracellular matrix deposition (panels D–F), Lipid deposition (panels G–I), Macrophages (panels J–L) and T lymphocytes (panels M–O). The amount of extracellular matrix, lipids, macrophages and T-lymphocytes is expressed as percentage of the stained area over the total plaque area. Bar in panel A = 100 µm. Positive area (%) refers to the percentage of the plaque area occupied by connective tissue, lipids, macrophages and T lymphocytes, respectively. Data are shown as means ± SD for 6 mice for each diet and unpaired *t*-test was used to detect significance (*P<0.05).

A significant reduction in lesion area was observed at the aortic sinus of mice fed SPH compared to controls (1.27±0.41×10^5^ µm^2^ vs. 2.02±0.31×10^5^ µm^2^; **Fig. 2A–C**). Plaque stability is an important factor concerning the severity of atherosclerosis. However, histological/immunohistochemical characterization of atherosclerotic lesions displayed no significant difference in plaque composition between mice fed SPH and controls, showing a comparable percentage of area occupied by extracellular matrix (34.56±0.56% vs. 30.31±18.25%; **Fig. 2D–F**), lipids (74.06±7.48% vs. 79.68±6.45%; **Fig. 2G–I**), macrophages (64.47±4.47% vs. 60.57±3.71%; **Fig. 2J–L**), and lymphocytes (27.36±11.73% vs. 22.62±7.24%; **Fig.** **2M–O**).

**Figure 3:**
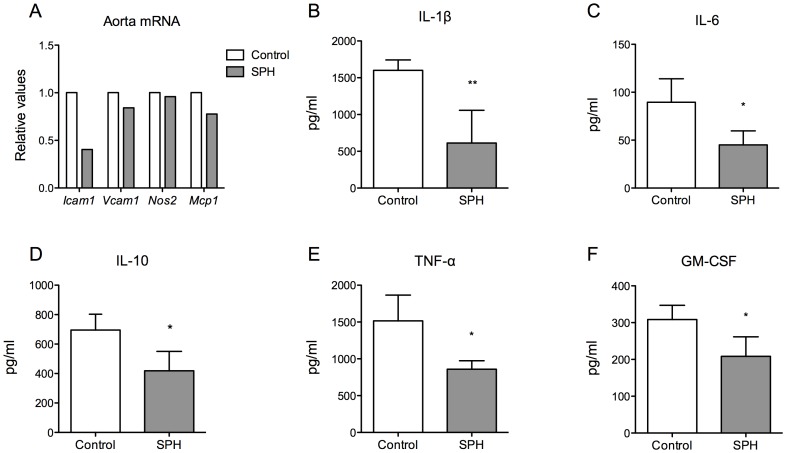
Levels of mRNA expression in aorta and inflammatory mediators in plasma in apoE^−/−^ mice fed a high-fat diet (control) or a diet with 5% SPH for 12 weeks. (A) The gene expressions of the inflammatory markers *Icam1*, *Vcam1*, *Nos2* and *Mcp1* were measured in pooled aortic arch from six mice. Inflammatory markers in blood samples collected at day 77 of treatment were analysed (B) IL-1β, (C) IL-6, (D) IL-10, (E) TNF-α, (F) GM-CSF and bars represent means ± SD of 4 pooled samples of 3 mice for each diet. Unpaired *t*-test was used to assess statistical significance and results significantly different from control are indicated (*P<0.05, **P<0.01).

Inflammation and oxidative stress are strong contributing factors in atherosclerosis, thus gene expression of inflammatory markers and redox regulators in aorta and heart were measured. Accompanied by decreased plaque area in sinus and aortic arch, mRNA level of intracellular adhesion molecule (*Icam1*) was decreased with 59.54%, in addition to a small decrease in expression of vascular cell adhesion molecule (*Vcam1*) and monocyte chemoattractant protein 1 (*Mcp1*) in pooled aortic arch from six mice, whereas mRNA level of inducible nitric oxidase 2 (*Nos2*) was not modified by the dietary treatment with SPH (**Fig. 3A**). In contrast, no changes were found in gene expression in the heart of *Icam1*, *Vcam1*, *Mcp1*, *Nos2* or *Tnfa*, nor of the antioxidant markers superoxide dismutase 1, soluble (*Sod1*), superoxide dismutase 2, mitochondrial (*Sod2*) or catalase (*Cat*) (data not shown).

### Decreased Plasma Levels of Inflammatory Markers

To further elucidate the potential anti-inflammatory effects of SPH in this experimental model of atherosclerosis, we examined plasma levels of inflammatory mediators. As shown in **Fig. 3B–F**, levels of IL-1β, IL-6, IL-10, TNF-α and GM-CSF were significantly lower in SPH-treated mice compared to controls.

### SPH-intervention Affected Hepatic mRNA Expression Involved in Lipogenesis

**Figure 4:**
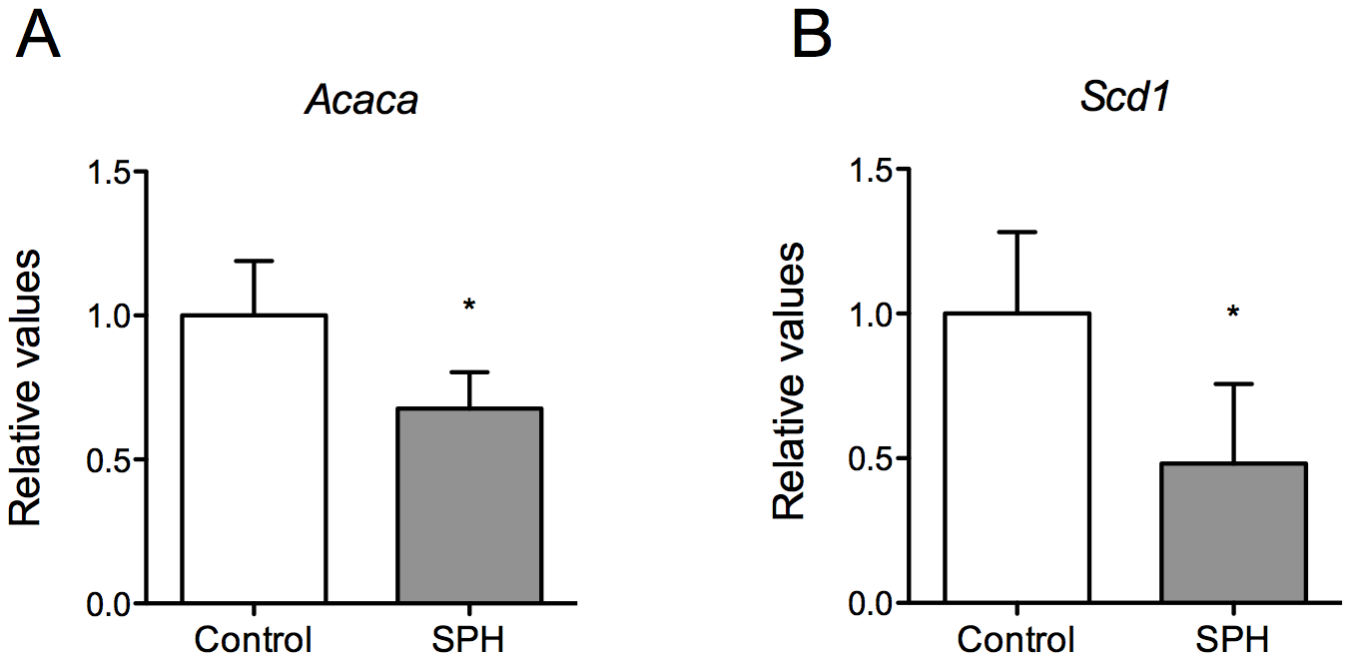
Hepatic gene expression in apoE^−/−^ mice fed a high-fat diet (control) or a diet with 5% SPH for 12 weeks. Hepatic mRNA levels of (**A**) *Acaca* and (**B**) *Scd1*. Data for gene expressions are shown as mean values relative to control ± SD for 4 mice for each diet. Mann-Whitney test was used to assess statistical significance (*P<0.05).

Hyperlipidemia is closely linked to atherosclerotic development. Liver is the main tissue regulating lipid metabolism, and mitochondrial β-oxidation is important in regulating plasma TAG. Hepatic gene expression showed a significant downregulation in mRNA level of *Acaca* in SPH-fed mice (**Fig. 4A**). Moreover, the mRNA level of *Scd1* was significantly downregulated as well (**Fig. 4B**).

Noteworthy, SPH administration had no effect on palmitoyl-CoA oxidation in the presence and absence of malonyl-CoA (**Fig. A** in **Table S1)**, nor on mitochondrial and peroxisomal fatty acid oxidation as the enzyme activities of CPT2 and ACOX1, respectively, were unchanged (**Fig. B** and **C** in **Figure S1**). ACAT activity, involved in cholesteryl ester synthesis, was also unaltered (**Fig. D** in **Figure S1**).

### Effects of SPH on Lipid Concentration and Fatty Acid Composition in Plasma

In order to evaluate the effect of SPH treatment on plasma lipid concentration, blood was collected for enzymatic measurement of lipid profile after 77 days of dietary treatment. As shown in **Table 1**, plasma total- and free-cholesterol, as well as TAG, cholesteryl esters and phospholipids concentrations displayed comparable levels between SPH-group and control group at the end of treatment period, whereas NEFAs increased in SPH-fed mice vs. controls (**Table 1**). Moreover, no difference was observed between the two groups in the relative amount of saturated fatty acids (SFA) (**Table 2**). The relative amount of monounsaturated fatty acids (MUFA) in SPH-fed mice was slightly lower than controls at day 77, mainly due to a small decrease in 18∶1n-9 (oleic acid) and 18∶1n-7 (vaccenic acid) (**Table 2**). Total n-6 PUFAs displayed a higher amount after 77 days of treatment in the SPH-group, probably due to the increase of C18∶2n-6 (linoleic acid) and C20∶4n-6 (arachidonic acid) compared to controls. In contrast, no differences were detected in the weight % of n-3 PUFAs between the two groups. As a consequence, a small reduction in n-3/n-6 ratio was observed after 77 days. Overall, the effect of the SPH-diet on plasma lipids and fatty acids was modest.

**Table 1:** Plasma lipids in apoE^−/−^ mice fed a high-fat casein diet (control) or a high-fat diet with 5% SPH after 77 days of dietary treatment.

1Lipid class	Day 77	
	Control	SPH
Cholesterol	12±0.9	11±1.0
TAGs	1.4±0.1	1.3±0.1
Phospholipids	3.0±0.1	3.0±0.1
NEFAs	0.8±0.2	1.1±0.1*
Cholesteryl esters	8.1±0.8	7.9±0.8
Free Cholesterol	3.7±0.1	3.4±0.2

1 mmol/L.

Data are shown as mean ± SD (*n* = 4).

Abbreviations: NEFA, non-esterified fatty acid; SPH, salmon protein hydrolysate; TAG, triacylglycerol.

*P<0.05 vs. control.

**Table 2:** Plasma fatty acid composition in apoE^−/−^ mice fed a high-fat casein diet (control) or a high-fat diet with 5% SPH after 77 days of dietary treatment.

1Fatty acids	Control	SPH
∑SFAs	32±0.5	34±0.5
∑MUFAs	31±0.4	30±0.4*
C18∶1n-9 (oleic acid)	25±0.4	24±0.5
C18∶1n-7 (vaccenic acid)	1.3±0.0	1.2±0.0*
n-6 PUFAs	28±0.4	30±0.4**
C18∶2n-6 (linoleic acid)	15±0.1	16±0.2***
C20∶4n-6 (arachidonic acid)	12±0.4	13±0.2*
n-3 PUFAs	6.4±0.3	6.3±0.3
C20∶5n-3 (eicosapentaenoic acid)	0.53±0.0	0.4±0.0
C22∶6n-3 (docosahexaenoic acid)	5.0±0.3	5.0±0.2
n-3/n-6	0.2±0.0	0.2±0.0*

1 Fatty acids (% w/w).

Data are shown as mean ± SD (*n* = 4).

Abbreviations: MUFAs, monounsaturated fatty acids; PUFAs, polyunsaturated fatty acids; SFAs, saturated fatty acids; SPH, salmon protein hydrolysate.

*P<0.05 vs. control.

**P<0.01 vs. control.

***P<0.001 vs. control.

## Discussion

Fish intake is inversely correlated to CVD-risk factors in both observational and clinical interventional trials [28]. Particular attention has been drawn to the cardio-protective effects of fatty fish species with high levels of omega-3 PUFAs through their lipid-lowering, anti-inflammatory, antiplatelet and antiarrhythmic mechanisms [29], [30]. Marine organisms are also a rich source of bioactive proteins and peptides that may induce health benefits through antihypertensive and antioxidative [28], immunomodulating [31] and lipid-lowering effects [14], [17]. Thus, marine proteins and peptides have been shown to influence the two major risks for atherosclerotic development, namely hyperlipidemia and inflammation. Therefore, it was of interest to investigate a potential anti-atherosclerotic effect of SPH-diet in apoE^−/−^ mice fed a high-fat diet. Although these mice spontaneously develop atherosclerosis on a standard rodent diet, a high-fat diet regimen, combined with female mice, was preferred to accelerate the progression. We showed that apoE^−/−^ mice fed a high-fat diet containing 5% (w/w) SPH for 12 weeks developed less atherosclerotic plaques compared to controls. In particular, we observed a significant reduction of plaque area in the aortic arch as well as in the aortic sinus. The pathophysiological complication of atherosclerosis is plaque rupture causing heart attack and stroke in humans. Vulnerability of plaque rupture is an important element in the fatal outcomes of atherosclerosis, and content and stability of the plaque is therefore of interest. However, there was no change in aortic sinus plaque composition of connective tissue, macrophages or lymphocytes, indicating that SPH had no effect on plaque stability. Unfortunately, apoE^−/−^ mice are not susceptible to the progress of plaque rupture unless treated with a high-fat diet for over a year, thus studying plaque stability in this model is limited.

During plaque development, accumulation of adhesion molecules contributes to foam cell formation. In addition to decreased plaque area in aortic arch, a decrease in expression of the adhesion molecule *Icam1*, as well as a small reduction in *Vcam1* and the chemokine *Mcp1*, was detected in pooled aortic arch of SPH-treated mice, suggesting a local anti-atherosclerotic effect of the SPH-diet. The plaque area decreased, but no reduction in number of macrophages was observed with immunostaining in the aortic sinus. This could be due to a simultaneous decrease in number of macrophages and plaque area, which would not be reflected in a percentage measurement. The mRNA level of inflammatory markers in heart was unaltered, and could explain the unchanged levels of macrophages. However, mRNA levels were measured in total heart that may weaken a potential reduction of these inflammatory markers. The decrease in sinus plaque area, without a change of macrophages could also be explained by shrinkage of the lipid-rich core due to fewer lipids, thus the macrophages decrease in size.

Liver is the main organ regulating lipoprotein metabolism, including plasma TAG and cholesterol levels, and a high dietary cholesterol intake has been reported to elevate liver inflammation [8]. Noteworthy, the plasma concentrations of cholesterol and TAG were not affected by SPH-treatment. This was accompanied by unchanged fatty acid oxidation and ACAT activity. These results are in contrast with previous reports showing cholesterol-lowering effects of fish protein hydrolysates in both rats and mice [14], [16]. Although gene expressions of *Acaca* and the Δ9-desaturase *Scd1* were decreased, it did not affect plasma TAG in apoE^−/−^mice. This lack of effect could be explained, at least partially, by the lower amount of fish protein used in the present study (5%) compared to previous studies, where 10–25% fish protein hydrolysate were applied [14], [16], [17]. In C57BL/6 mice fed 5% SPH for 6 weeks, a 32% decrease in plasma TAG has been found, but no change in plasma cholesterol (data to be published). Thus, in the present study, the disturbed plasma lipid transport in the apoE^−/−^ mouse model might have interfered with the potential TAG-lowering mechanism of SPH, while cholesterol-lowering effect might not be expected at this dose. A lower cholesterol level has been observed in animal studies when taurine was added in the diets [32], [33]. However, in our study, the cholesterol level was not affected after intervention despite the presence of taurine in the SPH-diet.

The plasma level of NEFAs was unchanged by SPH administration and only minor alterations were observed in plasma fatty acid composition. During the 12 weeks of feeding the plasma level of MUFAs was slightly lower in the SPH-fed group, but this was probably not of biological significance. Total n-6 PUFAs in plasma was higher in SPH-fed mice at the end-point measurement. Arachidonic acid and oleic acid was increased and decreased in the SPH group and controls, respectively, after the feeding period. The increase in arachidonic acid and linoleic acid with a simultaneously decrease in oleic acid might be due to increased synthesis of arachidonic acid and linoleic acid from their precursor oleic acid. Although arachidonic acid is considered pro-inflammatory [34], we detected reduction in plaque area in aortic arch and sinus, suggesting that SPH reduced atherosclerotic activity independent of the plasma arachidonic acid level. n-3 PUFAs, the n3/n6 ratio and anti-inflammatory index were not affected by SPH feeding, which is in contrast to previous findings [35]. However, as stated previously, in the current study we used a smaller amount of fish protein (5% vs. 15%) and the mouse model could also influence the effect on fatty acid composition. Knockout of the apoE gene causes an abnormal plasma lipid composition and metabolism, which apparently this SPH-diet cannot counteract.

Cytokines play a key role in the progression of atherosclerosis and it was of interest to note that the reduction in plaque area in the aortic arch was accompanied by a lowering of inflammatory markers in plasma, as reported in another study using salmon protein on inflammatory bowel disease in rats [18]. Peroxisome proliferator-activated receptors (PPAR), which are ligand-dependent transcriptional factors regulating both fatty acid [36] and amino acid metabolism [37], are shown to exert anti-inflammatory potential by inhibiting expression of cytokines and other pro-inflammatory factors [38]. The mechanism is unclear, but Zhu *et al.* has recently shown that marine peptides may act as PPAR-agonists and exert an anti-inflammatory effect [39]. Altogether, these results suggest that SPH administration might prevent atherosclerotic development by inhibiting activation of systemic inflammation.

A small dose of SPH 3.5% in rats has been shown to potentially exert antioxidant activities by reducing markers for oxidative stress in colon [18]. In the current study, gene expressions of the antioxidants *Sod1*, *Catalase* and *Nos2* in the heart were unchanged by SPH administration, suggesting that SPH did not affect the antioxidant defence system in the heart of apoE^−/−^ mice.

Although the present study has some limitations, such as absent protein data on inflammatory mediators within the aortic lesions, it gives indication that a salmon protein source may have a protective role in atherosclerotic development through mechanisms linked to inhibition of inflammation, and not directly related to plasma lipid changes. Although the apoE^−/−^ mice model has been used extensively in experiments studying atherosclerosis as it gives the opportunity to study genetic influence on atherosclerosis without using a high-fat diet rich in cholesterol, it is also a challenging model to use. These mice develop severe atherosclerosis due to accumulation of VLDL in plasma carrying most of the cholesterol. VLDL, containing apoB-48, is considered more atherogenic than the apoB-100-containing LDL. High plasma levels of LDL are also most present in humans with atherosclerosis, therefore in future studies it would be of interest to test this SPH in LDLr^−/−^ mice.

## Supporting Information

Figure S1 Hepatic enzyme activities of enzymes involved in peroxisomal and mitochondrial β-oxidation; (**Figure A**) Palmitoyl-CoA-β-oxidation with and without inhibition with malonyl-CoA, (**Fig.** **B**) CPT2 activity, (**Fig.** **C**) ACOX1 activity and (**Fig.** **D**) ACAT activity.
(TIFF)

Table S1 Composition and amino acid contents of the diets.
(DOCX)

Table S2 Overview of analysed genes.
(DOCX)

## References

[1] Lloyd-Jones DM (2010) Cardiovascular risk prediction: basic concepts, current status, and future directions. Circulation 121: 1768–1777. 20404268 10.1161/CIRCULATIONAHA.109.849166

[2] Woollard KJ (2013) Immunological aspects of atherosclerosis. Clinical Science 125: 221–235. 23668229 10.1042/CS20120576

[3] Hansson GK, Robertson A-KL, Söderberg-Nauclér C (2006) Inflammation and atherosclerosis annual review of pathology: Mechanisms of disease. Annual Reviews 1 297–329. 10.1146/annurev.pathol.1.110304.100100 18039117

[4] van der Wal AC, Becker AE, van der Loos CM, Das PK (1994) Site of intimal rupture or erosion of thrombosed coronary atherosclerotic plaques is characterized by an inflammatory process irrespective of the dominant plaque morphology. Circulation 89: 36–44. 8281670 10.1161/01.cir.89.1.36

[5] Sabesin SM (1981) Lipid and lipoprotein abnormalities in alcoholic liver disease. Circulation 64: III 72–84. 7020988

[6] Kleemann R, Kooistra T (2005) HMG-CoA reductase inhibitors: effects on chronic subacute inflammation and onset of atherosclerosis induced by dietary cholesterol. Curr Drug Targets Cardiovasc Haematol Disord 5: 441–453. 16503864 10.2174/156800605774962077

[7] Rein D, Schijlen E, Kooistra T, Herbers K, Verschuren L, et al (2006) Transgenic flavonoid tomato intake reduces C-reactive protein in human C-reactive protein transgenic mice more than wild-type tomato. J Nutr 136: 2331–2337. 16920850 10.1093/jn/136.9.2331

[8] Kleemann R, Verschuren L, van Erk MJ, Nikolsky Y, Cnubben NH, et al.. (2007) Atherosclerosis and liver inflammation induced by increased dietary cholesterol intake: a combined transcriptomics and metabolomics analysis. Genome Biol 8 R200. 10.1186/gb-2007-8-9-r200 PMC2375038 17892536

[9] Singh U, Jialal I (2006) Oxidative stress and atherosclerosis. Pathophysiology 13: 129–142. 16757157 10.1016/j.pathophys.2006.05.002

[10] Ridker PM, Rifai N, Cook NR, Bradwin G, Buring JE (2005) Non-HDL cholesterol, apolipoproteins A-I and B100, standard lipid measures, lipid ratios, and CRP as risk factors for cardiovascular disease in women. JAMA 294: 326–333. 16030277 10.1001/jama.294.3.326

[11] Harnedy PA, FitzGerald RJ (2012) Bioactive peptides from marine processing waste and shellfish: A review. Journal of Functional Foods 4: 6–24.

[12] Chalamaiah M, Dinesh Kumar B, Hemalatha R, Jyothirmayi T (2012) Fish protein hydrolysates: proximate composition, amino acid composition, antioxidant activities and applications: a review. Food Chem 135: 3020–3038. 22980905 10.1016/j.foodchem.2012.06.100

[13] Elvevoll EO, Eilertsen KE, Brox J, Dragnes BT, Falkenberg P, et al (2008) Seafood diets: hypolipidemic and antiatherogenic effects of taurine and n-3 fatty acids. Atherosclerosis 200: 396–402. 18242615 10.1016/j.atherosclerosis.2007.12.021

[14] Liaset B, Madsen L, Hao Q, Criaeles G, Mellgren G (2009) Fish protein hydrolysate elevates plasma bile acids and reduces visceral adipose tissue mass in rats. Biochim Biophys Acta 1791: 254–262. 19416649 10.1016/j.bbalip.2009.01.016

[15] Jacques H, Gascon A, Bergeron N, Lavigne C, Hurley C, et al.. (1995) Role of dietary fish protein in the regulation of plasma lipids. Can J Cardiol 11: Suppl G 63G–71G. 7585295

[16] Zhang X, Beynen AC (1993) Influence of dietary fish proteins on plasma and liver cholesterol concentrations in rats. Br J Nutr 69: 767–777. 8329352 10.1079/bjn19930077

[17] Wergedahl H, Liaset B, Gudbrandsen OA, Lied E, Espe M, et al (2004) Fish protein hydrolysate reduces plasma total cholesterol, increases the proportion of HDL cholesterol, and lowers acyl- CoA:cholesterol acyltransferase activity in liver of Zucker rats. Journal of Nutrition 134: 1320–1327. 15173391 10.1093/jn/134.6.1320

[18] Grimstad T, Bjorndal B, Cacabelos D, Aasprong OG, Omdal R, et al (2012) A salmon peptide diet alleviates experimental colitis as compared with fish oil. Journal of Nutritional Science 1: 1–8. 10.1017/jns.2012.23 PMC4153328 25191568

[19] Palinski W, Ord VA, Plump AS, Breslow JL, Steinberg D, et al (1994) ApoE-deficient mice are a model of lipoprotein oxidation in atherogenesis. Demonstration of oxidation-specific epitopes in lesions and high titers of autoantibodies to malondialdehyde-lysine in serum. Arterioscler Thromb 14: 605–616. 7511933 10.1161/01.atv.14.4.605

[20] Bjorndal B, Vik R, Brattelid T, Vigerust NF, Burri L, et al (2012) Krill powder increases liver lipid catabolism and reduces glucose mobilization in tumor necrosis factor-alpha transgenic mice fed a high-fat diet. Metabolism 61: 1461–1472. 22538117 10.1016/j.metabol.2012.03.012

[21] Vigerust NF, Bjorndal B, Bohov P, Brattelid T, Svardal A, et al (2012) Krill oil versus fish oil in modulation of inflammation and lipid metabolism in mice transgenic for TNF-alpha. Eur J Nutr 52: 1315–1325. 22923017 10.1007/s00394-012-0441-2

[22] Strand E, Bjorndal B, Nygard O, Burrin L, Berge C, et al (2012) Long-term treatment with the pan-PPAR agonist tetradecylthioacetic acid or fish oil is associated with increased cardiac content of n-3 fatty acids in rat. Lipids in Health and Disease 11: 82. 22738017 10.1186/1476-511X-11-82 PMC3459737

[23] Berge RK, Flatmark T, Osmundsen H (1984) Enhancement of long-chain acyl-CoA hydrolase activity in peroxisomes and mitochondria of rat liver by peroxisomal proliferators. Eur J Biochem 141: 637–644. 6146524 10.1111/j.1432-1033.1984.tb08239.x

[24] Bremer J (1981) The effect of fasting on the activity of liver carnitine palmitoyltransferase and its inhibition by malonyl-CoA. Biochim Biophys Acta 665: 628–631. 7295757 10.1016/0005-2760(81)90282-4

[25] Skorve J, al-Shurbaji A, Asiedu D, Bjorkhem I, Berglund L, et al (1993) On the mechanism of the hypolipidemic effect of sulfur-substituted hexadecanedioic acid (3-thiadicarboxylic acid) in normolipidemic rats. J Lipid Res 34: 1177–1185. 8371065

[26] Madsen L, Rustan AC, Vaagenes H, Berge K, Dyroy E, et al (1999) Eicosapentaenoic and docosahexaenoic acid affect mitochondrial and peroxisomal fatty acid oxidation in relation to substrate preference. Lipids 34: 951–963. 10574660 10.1007/s11745-999-0445-x

[27] Field FJ, Albright E, Mathur S (1991) Inhibition of acylcoenzyme A: cholesterol acyltransferase activity by PD128O42: effect on cholesterol metabolism and secretion in CaCo-2 cells. Lipids 26: 1–8. 1675757 10.1007/BF02544016

[28] Cinq-Mars DC, Hu C, Kitts DD, Li-Chan EC (2008) Investigations into inhibitor type and mode, simulated gastrointestinal digestion, and cell transport of the angiotensin i-converting enzyme-inhibitory peptides in pacific hake (merluccius productus) fillet hydrolysate. J Agric Food Chem 56: 410–419. 18163568 10.1021/jf072277p

[29] Raatz SK, Silverstein JT, Jahns L, Picklo MJ (2013) Issues of fish consumption for cardiovascular disease risk reduction,. Nutrients 5: 1081–1097. 23538940 10.3390/nu5041081 PMC3705336

[30] Jung UJ, Torrejon C, Tighe AP, Deckelbaum RJ (2008) n-3 Fatty acids and cardiovascular disease: mechanisms underlying beneficial effects. Am J Clin Nutr 87: 2003S–2009S. 18541602 10.1093/ajcn/87.6.2003S

[31] Duarte J, Vinderola G, Ritz B, Perdigon G, Matar C (2006) Immunomodulating capacity of commercial fish protein hydrolysate for diet supplementation. Immunobiology 211: 341–350. 16716803 10.1016/j.imbio.2005.12.002

[32] Murakami S, Kondo-Otha Y (1998) Tomisawa (1998) Improvement in cholesterol metabolism in mice given chronic treatment of taurine and fed a high-fat diet. Life Sciences 64: 83–91. 10.1016/s0024-3205(98)00536-0 10027745

[33] Chen W, Matuda K, Nishimura N, Yokogoshi H (2004) The effect of taurine on cholesterol degradation in mice fed a high-cholesterol diet. Life Sciences. 74: 1889–1898. 10.1016/j.lfs.2003.08.041 14761670

[34] Dembinska-Kiec A, Gryglewski RJ (1986) Contribution of arachidonic acid metabolites to atherosclerosis. Wien Klin Wochenschr 98: 198–206. 3010578

[35] Bjorndal B, Berge C, Ramsvik MS, Svardal A, Bohov P, et al (2013) A fish protein hydrolysate alters fatty acid composition in liver and adipose tissue and increases plasma carnitine levels in a mouse model of chronic inflammation. Lipids Health Dis 12: 143. 24098955 10.1186/1476-511X-12-143 PMC4021737

[36] Muoio DM, Way JM, Tanner CJ, Winegar DA, Kliewer SA, et al (2002) Peroxisome proliferator-activated receptor-alpha regulates fatty acid utilization in primary human skeletal muscle cells. Diabetes 51: 901–909. 11916905 10.2337/diabetes.51.4.901

[37] Kersten S, Mandard S, Escher P, Gonzalez FJ, Tafuri S, et al (2001) The peroxisome proliferator-activated receptor alpha regulates amino acid metabolism. FASEB J 15: 1971–1978. 11532977 10.1096/fj.01-0147com

[38] Delerive P, Fruchart JC, Staels B (2001) Peroxisome proliferator-activated receptors in inflammation control. J Endocrinol 169: 453–459. 11375115 10.1677/joe.0.1690453

[39] Zhu CF, Li GZ, Peng HB, Zhang F, Chen Y, et al (2010) Treatment with marine collagen peptides modulates glucose and lipid metabolism in Chinese patients with type 2 diabetes mellitus. Appl Physiol Nutr Metab. 36: 797–804. 10.1139/H10-075 21164551

