# Supplementary figures and images for: A Salmon Protein Hydrolysate Exerts Lipid-Independent Anti-Atherosclerotic Activity in ApoE-Deficient Mice
Source: PLoS One. 2014 May 19;9(5):e97598. doi: 10.1371/journal.pone.0097598 (PMC4026378; doi:10.1371/journal.pone.0097598)

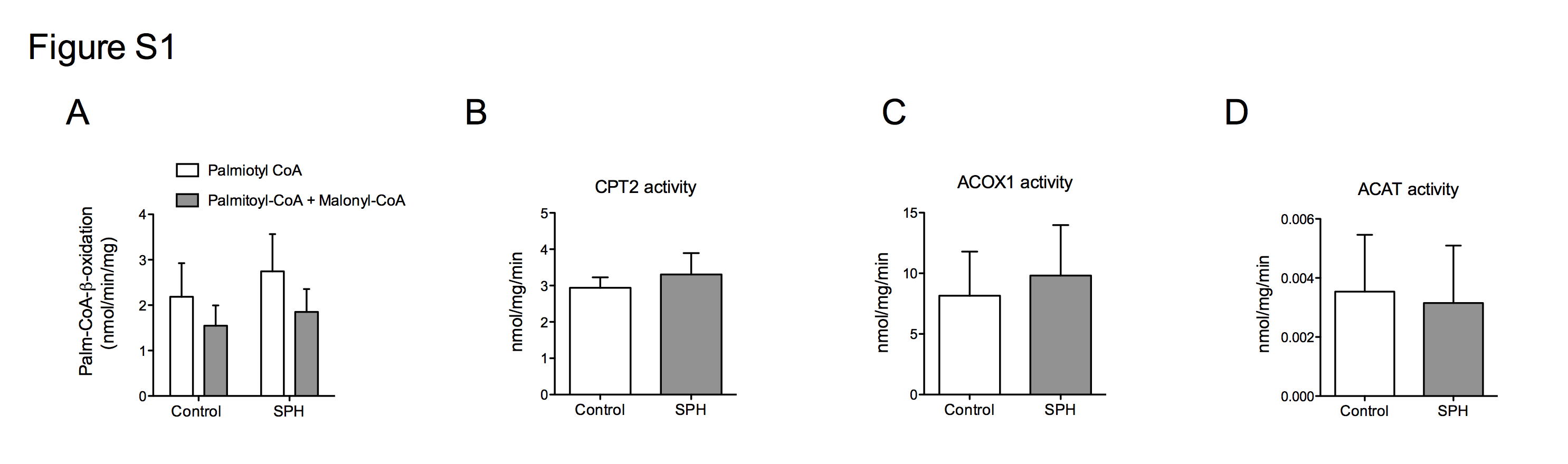

Supplement: Figure S1 — Hepatic enzyme activities of enzymes involved in peroxisomal and mitochondrial β-oxidation; (Figure A) Palmitoyl-CoA-β-oxidation with and without inhibition with malonyl-CoA, (Fig. B) CPT2 activity, (Fig. C) ACOX1 activity and (Fig. D) ACAT activity. (TIFF) [file pone.0097598.s001.tiff]
