# Supplementary material for: A Salmon Protein Hydrolysate Exerts Lipid-Independent Anti-Atherosclerotic Activity in ApoE-Deficient Mice
Source: PLoS One. 2014 May 19;9(5):e97598. doi: 10.1371/journal.pone.0097598 (PMC4026378; doi:10.1371/journal.pone.0097598)
Supplement: Table S1 — Composition and amino acid contents of the diets. (DOCX) [file pone.0097598.s002.docx]

| **Table S1** | | |
| --- | --- | --- |
| ^1^Composition of the diets | | |
| ^2^**Ingredients** | ^3^**Control** | ^4^**SPH** |
| *Protein source* |  |  |
| Casein | 250 | 200 |
| Salmon protein hydrolysate | 0 | 50 |
| *Fat source* |  |  |
| Soy oil | 24 | 24 |
| Lard | 213 | 213 |
| ^5^**Amino acid composition** |  |  |
| Hydroxyproline | 0 | 0.4 |
| Histidine | 6.4 | 5.4 |
| Taurine | 0 | 0.8 |
| Serine | 13 | 11 |
| Arginine | 7.5 | 7.1 |
| Glycine | 4.2 | 5.4 |
| Aspartic acid | 17 | 15 |
| Glutamic acid | 51 | 44 |
| Threonine | 9.4 | 8.3 |
| Alanine | 6.8 | 7.3 |
| Proline | 24 | 20 |
| Lysine | 18 | 17 |
| Tyrosine | 9.8 | 7.4 |
| Methionine | 6.0 | 5.3 |
| Valine | 14 | 13 |
| Isoleucine | 11 | 9.7 |
| Leucine | 21 | 19 |
| Phenylalanine | 12 | 9.0 |
| SPH, salmon protein hydrolysate | | |
| ^1^The diets were isonitrogenous, and contained 21 g protein per 100 g | | |
| diet | | |
| ^2^Ingredients (g/kg diet) | | |
| ^3^Amino acid composition (mg/g protein) | | |
| ^4^Casein consisted of 84% protein and 0.2% fat | | |
| ^5^SPH consisted of 91% protein | | |
| Other ingredients (g/kg diet): Cornstarch (105); Dyetrose (154); | | |
| Sucrose (117); Fiber (58); Dextrin/Cellulose (20); AIN-93G-MX mineral | | |
| mix (41); AIN-93G-VX vitamin mix (12); L-Cysteine (3.5); Choline | | |
| bitartrate (17); *tert*-Butyl-hydroquinone (0.016) | | |
