# Supplementary material for: A Salmon Protein Hydrolysate Exerts Lipid-Independent Anti-Atherosclerotic Activity in ApoE-Deficient Mice
Source: PLoS One. 2014 May 19;9(5):e97598. doi: 10.1371/journal.pone.0097598 (PMC4026378; doi:10.1371/journal.pone.0097598)
Supplement: Table S2 — Overview of analysed genes. (DOCX) [file pone.0097598.s003.docx]

| **Table S2** | | | | | |
| --- | --- | --- | --- | --- | --- |
| Gene names of analysed genes, primer/probe sets and analysed tissues | | | | | |
| Gene | Full name | ^1^AoD no. | Liver | Heart | Aorta |
| *Acaca* | Acetyl-Coenzyme A carboxylase alpha | Mm01304277_m1 | x |  |  |
| *Scd1* | Stearoyl-Coenzyme A desaturase 1 | Mm00772290_m1 | x |  |  |
| *Cat* | Catalase | Mm00437992_m1 |  | x |  |
| *Mcp1* | Monocyte chemoattractant protein | Mm00441242 |  | x | x |
| *Icam1* | Intracellular adhesion molecule | Mm00516023_m1 |  | x | x |
| *Nos2* | Nitric oxide synthase 2 | Mm00440502_m1 |  | x | x |
| *Sod1* | Superoxide dismutase 1, soluble | Mm01344233_g1 |  | x |  |
| *Sod2* | Superoxide dismutase 2, mitochondrial | Mm01313000_m1 |  | x |  |
| *Tnfa* | Tumor necrosis factor alpha | Mm00443260_g1 |  | x |  |
| *Vcam1* | Vascular cell adhesion molecule 1 | Mm00443281 |  | x | x |
| ^1^ Catalog number of custom TaqMan gene expression primer/probe sets from Applied Biosystems. | | | | | |
